# 3D printing of pharmaceuticals: approach from bench scale to commercial development

**DOI:** 10.1186/s43094-022-00439-z

**Published:** 2022-11-26

**Authors:** Ranjitsinh Pawar, Atmaram Pawar

**Affiliations:** Department of Pharmaceutics, Poona College of Pharmacy, Bharati Vidyapeeth (Deemed to Be University), Pune, Maharashtra 411038 India

**Keywords:** Additive manufacturing, Automation, Computer drug design, Fused deposition modeling, Personalized medicines

## Abstract

**Background:**

The three-dimensional (3D) printing is paradigm shift in the healthcare sector. 3D printing is platform technologies in which complex products are developed with less number of additives. The easy development process gives edge over the conventional methods. Every individual needs specific dose treatment. ‘One size fits all’ is the current traditional approach that can shift to more individual specific in 3D printing. The present review aims to cover different perspectives regarding selection of drug, polymer and technological aspects for 3D printing. With respect to clinical practice, regulatory issue and industrial potential are also discussed in this paper.

**Main body:**

The individualization of medicines with patient centric dosage form will become reality in upcoming future. It provides individual’s need of dose by considering genetic profile, physiology and diseased condition. The tailormade dosages with unique drug loading and release profile of different geometrical shapes and sizes can easily deliver therapeutic dose. The technology can fulfill growing demand of efficiency in the dose accuracy for the patient oriented sectors like pediatric, geriatric and also easy to comply with cGMP requirements of regulated market. The clinical practice can focus on prescribing each individual’s necessity of dose.

**Conclusion:**

In the year 2015, FDA approved first 3D printed drug product, which is initiator in the new phase of manufacturing of pharmaceuticals. The tailormade formulations can be made in future for personalized medications. Regulatory approval from agencies can bring the 3DP product into the market. In the future, formulators can bring different sector-specific products for personalized need through 3DP pharmaceutical product.

**Graphical Abstract:**

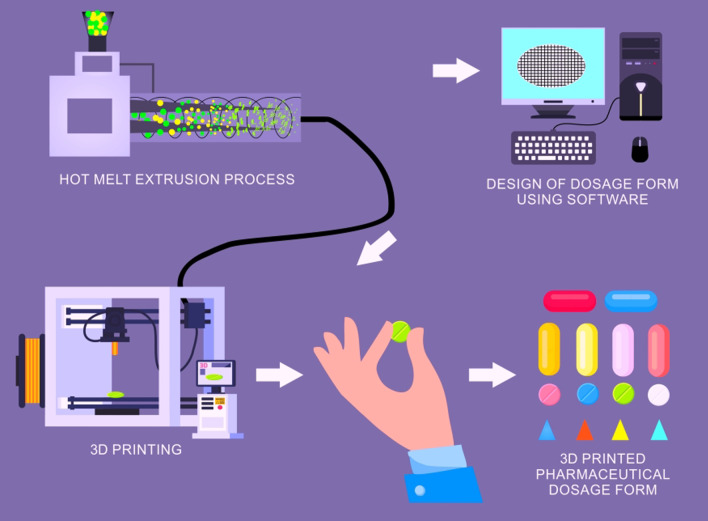

## Background

3D printing technology has been used in a variety of applications for over 30 years, including automotive, robotics, prototyping and chemical sciences [1]. This new industrial revolution will have a huge impact on the pharmaceutical industry. The pharmaceutical industry is currently using traditional methods to accept products into the market. 3D printing is the process of adding layers one on the other by using 3D printer. Because of the additional layers, it is also known as additive manufacturing. A file is created in a computer with all parameters such as infill percentage, geometry, printing speed, layer height, thickness, extrusion and platform temperature in the design by software and it is sent to a printer in.stl format for printing where the desired product can be printed using the compatible excipients. 3D printing is a tailored formulation development technology that can cater to the needs of each individual patient. One of the most important factors in targeted drug delivery is administration dose accuracy. Currently, the industry follows a one-size-fits-all approach; however, patient-specific dosage formulation can replace standard mass production of doses [2].

Personalized medication is a patient-specific approach to treating anyone with high precision and accuracy in dose. When compared to other dosage forms, it has a high precision, making it a patient-centric dosage formulation [3]. Individualization of medication is required due to variations in patients' physiological factors such as age, weight, and current body conditions. The specific diseased condition aimed at a specific population group can be easily prepared for prototype formulation. The formulation is created with the individual's essential dose in mind. If a slightly higher dose than required is used, 3D printing can help to reduce the side effects that are common in standard dosage form. It was discovered in the study that improper dose and its combinations caused 75–85% of the adverse conditions [4].

Oral administration has advantages over other dosage forms, tablet is a highly accepted dosage form among various industrial available dosage forms. 3D printing is also used in cosmetics and nutraceuticals. This technique can easily treat different people's skin; skin and hair problems can be diagnosed and treated by providing individuals with special tailor-made formulations. Skin sensitivity is an important factor to consider when treating with medications in cosmeceuticals. Safety and precautions can be taken as needed by each individual in order to address the problems of each patient. Similarly, nutraceuticals benefit each individual by providing the necessary nutrition supplement. 3D printing was also useful in dental applications such as oral and maxillofacial surgery; it can easily prepare complex structures such as the maxilla, mandible, skull, and dental prostheses [5, 6]. In the case of maxillofacial defects, it can be prepared to meet the individual's needs for anatomical uniformity, tissue appearance, and tissue function [7]. Tissue engineering via 3D printing is one of the best solutions for dealing with issues such as organ failure and organ replacement. Approximately 90% of hearing devices are manufactured using the 3D printing method. It contributes to a more personalized process manufacturing scenario [8].

**Fig. 1:**
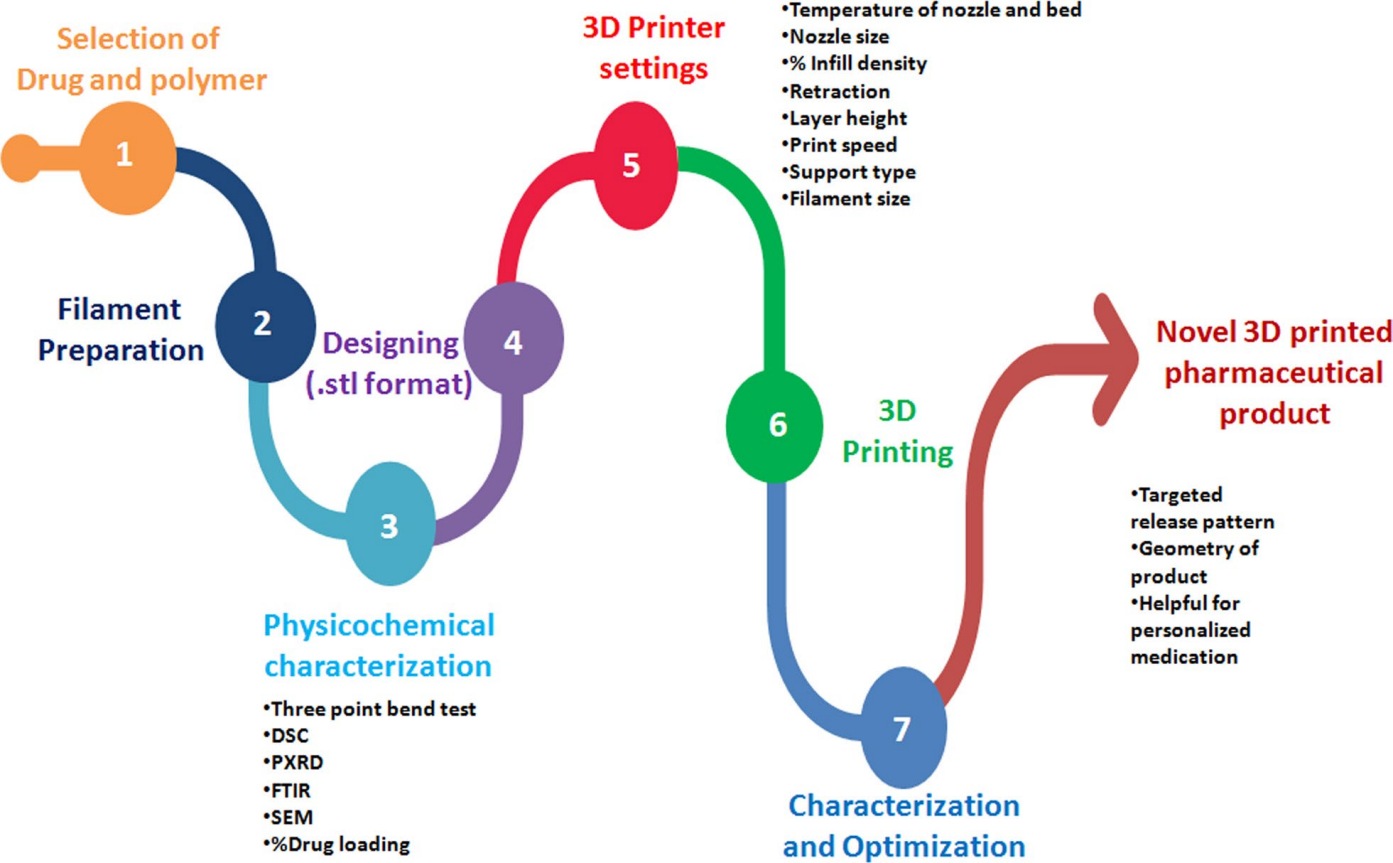
Stepwise process from drug selection to 3D printed product

In the case of cancer, 3D printing comes in handy because it is difficult to reach the specific tumor site. Because chemotherapeutic agents have low aqueous solubility during intravenous or oral drug administration, acting at the tumor site is not possible. In such a complex process, 3D printing can succeed. Recently, a 5-fluorouracil patch made of polylactic coglycolic acid and polycaprolactone was successfully printed. It is implanted directly into pancreatic cancer sites. After four weeks of effective treatment, the drug release becomes steady and constant. With the help of the patch, a total of four weeks of drug release kinetics are maintained throughout. Afterwards, it gets easily biodegraded inside the body [9]. In overall health scenario, improvement in healthcare sector helps to increase life expectancy and quality of human health with the newer techniques in the market (Fig. 1).

For acceptance in future making specific protocol for personalized medication will help to set up the regulatory guidelines [10]. Regulators from USFDA had taken interest in such advancement to give more specific guidelines to follow [11]. The potential of 3D printing industry is still unleash. In the upcoming decade, it can be fully evolved and emerge further. This review will provide a clear and comprehensive idea about the exciting new technology with different perspectives broadly [12].

## Main body

### Methodology

The elaborative bibliographic research was conducted so as to get clear idea about the development of 3D printing technology. We explored various literature searches by using different databases and search engines like PubMed, SciFinder, Scopus, Google scholar and Science Direct. This review article covers different approach about 3D printing. It includes 92 research articles and peer-reviewed articles. But it does not include abstract, conference communications and unpublished dissertations. All articles carefully chosen as per title and abstracts for the review.

### Techniques

In the development of 3D printing technology, newer advancements are made to prepare products as per the need of patient. In this section, we discuss different approaches to make 3D printed product. In traditional process, product is removed from solid blocks, after process of milling; hence, it is called subtractive process of manufacturing. In case of 3D printing, the object is made up of forming layer over each other. Hence, 3D printing also often called as ‘Additive manufacturing’.

#### Powder bed printing

In this technique, liquid solution of binder is sprayed over the thin layer of powder to adhere together by the smaller powder particles. It gets layered over each other; later on, excess powder can be removed by wipe off. But it is not made any hollow structure as that of other techniques like FDM. The first approved product is made by this technique. It adapts commercially with higher dose of range 250–1000 mg, for orally dispersible tablet (Table 1).

**Table 1:** Different drug-polymer combination with novelty described in 3D printing formulation

Sr. No	Active pharmaceutical ingredient	Polymer	Novelty in formulation	References
1	Fluorescein	PVA	The drug load is 0.29% with strong appearance of tablet. No drug degradation with modified release profile	[30]
2	Pramipexole	Eudragit EPO PEO	Drug release occurred within 5 min. No use of any disintegrating/filling agents	[60]
3	Theophylline	PVP	Reduce temperature and prepared IR tablet with talc	[61]
4	Pantoprazole sodium	PEG 6000 PVP K 12	Thermo-sensitive drug processed below 100^0^ C. Infill % lowers down to 50% help to reduce drug release up to 3 min	[62]
5	Paracetamol	PVP K12 and CCS	Oral-shaped tablet, Considering high dose prepared specially for drug release more than 90% within first 10 min	[63]
6	Paracetamol	PVA	4% drug load, different geometrical shapes are used such as pyramid, torus, cube, sphere, cylinder by keeping surface area constant	[32]
7	Hydrochlorothiazide	Eudragit E	Channeled tablet design. Increase surface area of tablet with media perforation through its structure. The shorter channels give more efficient drug release than the longer channels	[65]
8	Prednisolone	PVA	High thermal stability and neutral nature. Flexible dose ellipse-shaped tablets for drug release over the 24 h	[43]
9	Metformin	PVA	Egg-shaped tablet giving immediate drug release. Prepared as model drug for abuse deterrent formulation. It is well proven that it cannot break easily	[66]
10	Isoniazid Rifampicin	PEO	Different compartments are made so as to release drug as per its requirement. Anti-tuberculosis drug used can slower release and absorption of drug	[68]
11	Theophylline	Eudragit RL, Eudragit RS	Thermostable drug. With polymer Eudragit RL gives immediate and Eudragit RS gives extended drug release. The caplet form gives ease in the swallowing	[40]
12	Ramipril	Kollidon VA 64 Kollidon 12 PF	Drug melting point is 109^0^C. No degradation observed due to use of proper combination. Choice of combination becomes important so as to overcome degradation problem due to thermal heating process	[70]

For bulk manufacturing of tablets, the binding solution spread over powder bed using conveyor belt where powder deposits layer over each other as per the requirement, bounded particles stay together and unbound particles recycled at the starting process [13]. The first breakthrough made in 3D printing process is approval of Spritam® by Aprecia Pharmaceuticals (Levetiracetam drug used for treatment of epilepsy) from the US-FDA. The technology used for it was the Zip Dose® technology i.e. powder bed deposition method.

#### Pharmaceutical inkjet printing

The conventional inkjet printing technology is used since decades for reproducing different images, texts with their colors. The ink term is used when color pigments are used. In case of pharmaceuticals, printing of drug solution is used.

Printheads droplet size with range from 2 to 500 pL with frequency more than 2 kHz is used. The droplet size is kept smaller so as to print accurate therapeutic dose on printer. The inkjet printing is possible with solvents of aqueous or organic type to print accurate drug deposition for medical device or in unit dosage form. About 0.2 μL solution administered as the therapeutic dose to needy patients.

Two technologies are used, namely thermal inkjet (TIJ) and piezoelectric (PE). In thermal inkjet (TIJ), capillaries come in contact with resist element. It pulse the electric voltage so as to raise the temperature that cause vaporization of liquid and expand the vapor bubble. Then, bubble collapses again and is filled with fresh bubble from reservoir. The temperature can rise up to 300 °C, bubble can expand up to 3–10 μs, droplet is expressed out at 10 ms^−1^, and droplet volumes of 2–180 pL are possibly achieved. Though temperature is high, it only withstands for milliseconds. Thus, the solution of proteins like human growth hormone and insulin is reported that it is not able to denature by TIJ [14].

The inkjet printing for pharmaceutical use is cheap and easily modified as per the need. Different variables are used to control parameters like droplet size and software functions for controlling volume of solution. In attempt with desktop inkjet printer, it was used to print aqueous salbutamol sulphate solution onto starch-based oral films. The different concentration is in the range of 5–40 μg/mL print as film with high accuracy and precision [15].

In piezoelectric method, piezoelectric material is mounted outside the capillary. As electric voltage passed from it, it gets deformed and ejected out the droplet. Again it goes back to start position to create vacuum to refill capillary. The range of size is from 30 to 500 pL droplets. The process is repetitive; hence, it is called as drop on demand printing. It deposits about 200 million droplets per centimeter for its use. It also used jet excipient polymers with drug. For thermolabile drug, it explored the use of solenoid valve. In a reported study, comixture of caffeine and loperamide drug was used where loperamide not crystallize on the any substrate but caffeine is partially crystallized [16]. Also inkjet printing was used to prepare pharmaceutical co-crystals from comixture of drugs [17].

The optimum personalized medication should be easy, affordable and precise enough to accept by higher number of patients. But solid form like tablet if it gets split during printing it varies in the drug content [18]. It changes the release pattern and bioavailability of active element. But inkjet printing deposits the drug solution in different concentration in variation in the tablet or film for the fulfillment of required dose [19].

In case of commercial aspect, inkjet printing can produce a higher number of doses as per the requirements. Due to its ability in variation of doses, it is possible to prepare dosage form as per the need of patients. In future, pharmacies inkjet printing can be suitable method to prepare dose for patients in higher number [20].

But discrepancies like deviation in dose printing are observed in some cases. For increasing amount of deposition on substrate, it places back to printer several times. It was observed that dose get varied than predicted, and it might be because of substrate contact with printer roller [15]. In another study, deviation was observed as 11.8%, 24.3% and 34.9% for the copy paper, acetates and oro-dispersible films, respectively. It argued that it may be due to printer head get damaged by multiple uses [21]. Also, in case of theophylline tablets printing gets varied with relative standard deviations (RSD) of ± 5.1%, ± 6.3 and ± 6.25 for copy paper, coated paper and PET films, respectively. It is above the limit ± 5% of for variation limit. The near-infrared spectroscopy can be used to quantify amount deposited by tablet prepared by inkjet printing method [22].

In case of printing speed, it is observed that 8 mg API takes 2 min to print with 5.08 cm × 1.27 cm size. Also, 16 mm × 26 mm rectangle row of five can take only few seconds for preparation [21]. In jetting of 8 mg fenofibrate on stent takes 6.5–7 min to print by MicroFab printer [23]. The technique is highly spontaneous, with short time it produces dose of higher number.

It also has advantage of transferring dose from one device to another like micronozzle to microneedle. The regenerative medicines are also another application of inkjet printing. Poly (d, l) lactide with simvastatin or paclitaxel onto coronary metal stents can be printed. It has wide applications for bacteria, cells and nanoparticles to use as regenerative medicines [24, 25].

In application for preparation of particles, the liquid particle was prepared by freezing with nitrogen when it comes in contact with the liquid droplets. The freeze drying removes the water from porous particles to make it ideal aerodynamic nature for its pulmonary delivery. The successful attempt made with production of terbutaline sulphate particles by this method [26, 27].

## Nozzle-based deposition systems

Nozzle based deposition technique involved in proper mixing of drugs with polymers to proceed on 3D printing. The challenges faced by the previously discussed technique can be overcome by this technique. The nozzle was used to extrude for producing 3D printed product by layering method. Fused Deposition Modeling **(**FDM) involves melting whereas Pressure-Assisted Microsyringes (PAM) doesn’t need melting of components [28].

### Fused deposition modeling (FDM)

In FDM the hot melt extruded filament is used as primer for printing where drug loaded filament is passed through the nozzle. The melting temperature of drug-polymer mixture and extrusion temperature determines successful printing of product. The thermoplastic polymers like polyvinyl alcohol, polylactic acid and acrylonitrile butadiene styrene (ABS) are used but ABS is not sufficient for use in medical field as it is non-biodegradable polymer [29]. The temperature used must be above the melting temperature of components. The parameters such as feed rate, nozzle diameter, pressure drop and thermal properties such as glass transition temperature (*T*_g_), density, thermal conductivity of components determines performance of printing.

The first attempt made to print fluorescein with PVA filaments by FDM technique [30]. It proved that infill density can be modified to obtain desired drug release pattern. In further progression 4-ASA and 5-ASA used for colon treatment. In that 4-ASA having thermolabile property it gets degraded while printing but 5-ASA thermally stable and successfully printed. It proves that FDM is suitable technique for the use of non thermolabile components [31].

The FDM also helps in the geometry of tablets which is impossible in other 3D printing techniques. It was successfully proved that geometrical shapes are possible such as pyramid, sphere and torus. The constant geometry with different shapes is possible by varying dimension of the filaments. Also, modification in drug loading depending upon the need of patients is possible. The drug release kinetics with certain target can be achievable with printer settings. It is one of the best techniques to prepare tailormade formulations [32].

Compared to powder bed technique it provides more resolution, able to prepare complex design to achieve higher drug accuracy. It also provides good mechanical strength to give different release profiles. The infill percentage can change the release pattern of the drug profile. However thermal property plays an important role to decide the extrudability of any compound therefore API’s may not be suitable for FDM printing [33].

The polypill is possible with various active drugs, depending upon its compatibility 2–5 drugs can be used in one matrix structure by 3D printing method. The multiple drugs can gives targeted release profiles by using different polymer and infill density. Goyanes et. al. proved that multilayer tablets are possible by 3D printing method. Paracetamol and caffeine gives drug release kinetics as per their layers and location in the multilayer region [34].

The impact of 3D printing by FDM is higher than any other method. Different complex geometry is possible with different suitable drug-polymers. For clinical practice in future it needs to improve its drawbacks like speed and quality for the development.

### PAM technology (pressure-assisted microsyringes)

The technology depends upon the pressure applied on the piston to form desired geometry. The extruder via syringe deposits semi-liquid and viscous material. It depends upon the viscoelasticity, viscosity and elastic limit to reproduce the technique. The technology advances with use in continuous flow with room temperature which is not possible with other techniques. But solvent used might be failed at stability of certain APIs and toxic to health for human use. The technique can be used for drug delivery, tissue printing and soft tissue scaffolds [28, 35].

In the attempt to control the chitosan drug release so as to develop polylactic-coglycolic acid/nano-hydroxyapatite (PLGA/nHA) scaffolds having recombinant human bone morphogenetic protein 2 (rhBMP-2) nano-sustained release carriers with tissue engineered bone for the repairing large jaw defects [36]. The chitosan which is a natural product obtained from crustaceans shells like crabs and shrimps. It is polysaccharide comprises of copolymers of glucosamine and N-acetylglucosamine obtained by the chitin partial deacetylation. This cationic polyamine reacts chemically with anionic systems. In case of chitosan it is considered as a nonirritant and nontoxic agent as it is biodegradable and biocompatible. It is further used in different dosage form such as films, beads, gels, microspheres, coatings for liposomes and tablets with applications in the controlled drug delivery systems like colonic drug delivery and mucoadhesive dosage forms and immediate drug release.

### Laser-based writing system

The first device designed in the year 1980 by Charles Hull called as ‘Stereolithography’ (SLA). In this technique, the laser is used to photopolymerize resin for preparation. The printer consists of ultraviolet; it form laser to project on resin which is photopolymerizable in nature. The UV rays are used to interact and release free radicals from it [28]. The first layer gets solidify and new layer is added, the process goes on until 3D printed product gets ready. The thickness can be dependent upon the UV light energy, it gets solidify eventually. The resin chosen plays a vital role into process of resin exposure with UV light. This has to be approved by regulatory agency to get approved for its safe human use [28, 33].

The laser is moved to make the layer one over the other. Tank providing resin as laser moves it solidifies the resin to form a solid base. Drug can be easily disperse or dissolve inside the resin before printing. The solid oral tablets can be made by this method. In the first attempt aspirin with polyethylene glycol diacrylate (PEGDA) resin was made [37]. It gives complete drug release in time of 3 h. As the resin is miscible with water, hydrogels can be made easily.

In case of SLA, drug and photopolymer resin is previously mixed to make solid base. Thermal process involvement is lesser compared to the other technique like FDM. Also loading of drug is low as compared to other techniques. Recently polymers developed are diethyl fumarate (DEF), poly (propylene fumarate) (PPF), poly (2-hydroxyethyl methacrylate) (pHEMA), dimethacrylate (DMA), poly ethylene glycol and Polyethylene glycol diacrylate (PEGDA) [33]. The SLA technique is well versed with 3D printing so as to widen the scope in bioengineering.

In selective laser sintering technique (SLS) the laser is moved on the powder bed. The heat generated in the laser melt the particles to fuse together. Similar to powder bed printing previously discussed it helps to form a solid bed over it. It doesn’t use any solvent hence widen the future scope to develop this technique used in solid dosage form [38].

### Polymer selection

Selecting polymer for 3D printing formulation is important factor so as to achieve different release pattern. The therapeutic need of patients depends upon different release patterns like immediate, modified or extended release. It can be achieved by using different types of polymer as per the requirement.

Selection of appropriate polymer as per the need is basic requirement for any 3D printing formulation. In case of FDM two methods used namely hot melt extrusion (HME) and impregnation [39]. The HME technique is widely used due to its drug loading capacity is higher than any other method [40, 41]. However, currently available HME polymers sometimes not compatible to extrudable further for FDM printing [42]. In impregnation method filament is dipped in an organic solution for certain period of time, hence it will accumulate drug loading into it, but it is less in amount. In case of immediate drug release dosage form Kollidon® VA64, is good choice. Water soluble polymer polyvinylpyrrolidone-vinyl acetate was proven to be helpful for making 3D printing formulation [30, 31, 43].

For choosing polymer of suitable kind the criteria of selection depends upon the melting point, drug-polymer compatibility and extrudability of polymer. Polymer can be helpful to achieve target release for functional activity. It gives edge over the other currently available formulation by performing more accurately by 3D printing. Selecting polymers like PVA, PCL and PLA having sufficient thermoplasticity to make 3D printing formulation. The proper grade has to be chosen for not just preparation of filament but also to make extrudable from 3D printer. For the improvement in the process of release system different plasticizers are used like Eudragit RL, RS and HPC for FDM 3D printing [43, 44].

#### Polyvinyl alcohol (PVA)

It is water soluble thermoplastic synthetic polymer having no odour and good mechanical properties. It is made by hydrolysis of polyvinyl acetate either full or partial way, by removing acetate group from it [45]. This process has impact on the properties like physical, chemical and mechanical. The melting point decided by degree of hydrolysis, if it is ranging from 180 ℃ having partially hydrolyzed to 220 ℃ fully hydrolyzed. The viscosity of polymer ranging between 3.4 and 52 mPa·s is partially hydrolyzed whereas 4 to 60 mPa·s is fully hydrolyzed [28, 45]. The degree of hydrolysis and polymerization of polyvinyl alcohol is lower and higher is the solubility in water made crystallization easier [45]. Molecular weight of any polymer is higher than its degree of hydrolization which is lower. The glass transition temperature (*T*_g_) of PVA is 85 ℃ and temperature of degradation is about 350–450 ℃.

The good biodegradability and its less adverse effects make it suitable for biomedical pharmaceutical applications [45]. It is one of the applications in 3D printing is to produce multiple layers by inkjet printing method. The printable ink with glycerin is used to avoid blocking in nozzle allowing for easy printing of product. Molecular weight decides the inkjet printing capacity depending upon viscosity of ink. Higher molecular weight didn’t accumulate any color and stable enough for six months period. But the lower molecular weight forms milky appearance resulting into unsuitable for inkjet printing [46].

Apart from application in inkjet printing, PVA is also successfully applied in FDM technique. Depending upon the factors like infill density ranging from 0 to 100% it defines the structure. The extruder speed, layer height and temperature nozzle are different parameters taken in the consideration while using FDM technique. In case of filament impregnation first it is dried and later printed as per the need [24]. The previous attempt proved that drug can be successfully loaded up to 10%. In one of the attempt by Goyanes et. al. paracetamol and caffeine successfully loaded drug by hot melt extrusion with PVA filaments. The drug loading within range 4–10%, as loading is lower drug release is also becomes decrease. In setting parameters for FDM is 100% infill, 200 ℃ temperature of extrusion and extruding speed of 90 mm/s [47].

#### Polylactic acid (PLA)

The biodegradable polymer is suitable for applications in medical fields such as drug delivery, regenerative medicines, stent applications and tissue engineering. It is accepted by the United States Food and Drug Administration (FDA) and considered as safe for use [48]. The different properties depend upon the process temperature, molecular weight and isomers ratio. The PLA having melting point 150–175 ℃; *T*_g_ = 55 ℃ [29, 48] and it is soluble in dioxane, methylene chloride, acetonitrile, 1, 1, 2-trichloroethane, dichloroacetic acid and chloroform. The solubility is poor in water, propylene glycol, alcohols like methanol, ethanol and unsubstituted hydrocarbons like hexane and heptanes. But when solvents heated to boiling temperature solubility increased rapidly [48].

The PLA has one effective application that it doesn’t cause any toxic or carcinogenic effect. Administration into human body it gets hydrolyzes into alpha hydroxylic acid. Later, it gets into tricarboxylic acid cycle and excreted out from the body. Also, rate of degradation is depending upon factors like molecular weight, stereochemistry and crystallinity. The PLA has slower rate of degradation making it sustain for longer time in vivo type [48].

Thermal degradation can possibly cause due to lactide formation, hydrolysis and oxidative scission of chains. The degradation process lose 5% polymer at 325 ℃ and at 500 ℃ doesn’t have any residue. Because of low cell affinity causes inflammatory response when it has comes in direct contact with biological fluids. It is brittle polymer as compared to other polymers [48]. Considering all the factors PLA becomes the good choice for 3D printing technique [35].

#### Polycaprolactone (PCL)

It is the hydrophobic semi-crystalline polymer with melting point 59–64 ℃ with *T*_g_-60 ℃. It has good solubility in benzene, toluene, cyclohexane, carbon tetrachloride, chloroform, dichloromethane and 2-nitropropane at room temperature. Also, low solubility observed in acetonitrile, acetone, ethyl acetate, 2-butanone, dimethylformamide and insoluble in diethyl ether, alcohol and petroleum ether [49]. It gets degraded in environment by bacteria and fungi not by human body because it doesn’t contain necessary enzymes for its biodegradation. The PCL is bioresorbable later on start the hydrolytic degradation.

The molecular weight, degradation conditions and degree of crystallinity are the deciding factors for homopolymer polycaprolactone [49]. As crystallinity increases molecular weight decreases. As compared to PLA, PGA the time of degradation becomes longer in case of PCL. It helps to deliver drug for longer period of time by extending degradation period by at least a year. The various factors like low melting point, solubility and blend compatibility make PCL applied in the biomedical applications like wound dressings [50], tissue engineering [51] and drug delivery systems [49].

In case of 3D printing it was studied for tablets loaded with polymeric nanocapsules made up of Eudragit RL100 and PCL produced by FDM printer. The temperature of extrusion used was at 110 ℃ for Eudragit and at 65 ℃ for PCL. The printing temperature of Eudragit filaments was at 170 ℃ and at 95 ℃ for PCL. The settings for the FDM printer were 90 mm/s extruding speed and 100% infill percentage along with Eudragit filaments with 50% infill percentage so as to prepare tablets. Polymer decides the drug loading and drug content available in the 3D printed tablet. The Eudragit tablets have a higher drug content as compared to polycaprolactone due to its swelling indices is high. The release profile is high in case of Eudragit compared to PCL tablets [52].

#### Polyphenylene sulfide (PPS)

It is asymmetrical rigid backbone chain consists of Para substituted phenylene rings and sulfur atoms. It is semi-crystalline material with thermoplastic property; it has high temperature stability, good dimensional stability, flame retardance and chemical resistance for easy processability. It also has the capacity for radiation resistance, aging resistance and nontoxicity [53, 54].

In other sectors like aerospace, electronics and automotive it also has wider applications. In 3D printing thermal process involved affecting mechanical properties depending upon the temperature and cross-linking of polymers [55]. In analysis by the Park et. al. [56] it was observed that in the range of 200–250 ℃; higher oxygen concentration and rise in temperature effectively increases cross linking of polymers.

In case of PPS the studies carried out towards the parameters of printing, composites and effect on warpage. The semi-crystalline thermoplastic PPS and polyether ketone analyzed the material properties like rheology and thermal properties. From this, printing parameters are selected for printing. In another study, elemental analysis is done for residual stresses observed during the process of cooling PPS and PP for variation in printing speed [57].

### Drug development in 3D printing formulation

The various formulations are made by the different strategies for the formulation. Mostly FDM (Fused deposition modeling) is widely used in 3D printing pharmaceutical formulations. The percentage drug loading depends upon the physiochemical properties of drug, polymer used and printing setup. The FDM is mainly well explored with different model drugs to well-established prototype for 3D printing solid dosage form like tablet.

Amongst all one of the first attempt for modified release formulation by using FDM method was made with the model drug fluorescein. PVA filament was used with fluorescein with the swelling of PVA in ethanolic drug solution. The drug loading achieved was 0.29% w/w by this method. Appearance of tablet was strong enough and no drug degradation was observed. The tablets of 10 mm diameter were printed by using 3D printer. By change in the infill percentage it shows modified release profiles [30].

#### Immediate release

Immediate release is giving quick onset of action within shorter time period. The making physical modifications with low dose model drug Pramipexole by using Eudragit® EPO and polyethylene oxide. The drug achieves complete release within 5 min by change in physical modification along with Hot melt Extrusion and FDM 3D printing. So, synergistic approaches of techniques avoid the use of other disintegrating or filling agents. Different infill percentages like 10%, 25%, 50% and 75% extruded effectively with the use Eudragit® EPO-POLYOX. After POLYOX concentration decrease it helps to deform matrix structure and makes tablet release rapidly. The thickness of tablets can be decreased and creating space in between them to increase drug release. It is able to increase the drug release rate and possibly up to 5 min [58].

In another attempt Theophylline or dipyridamole loaded with PVP filament. For PVP filament ideal temperature for extrusion is ranging between 200 and 220 ℃ but in this case it extrudes easily at 110 ℃. In spite of hydrophilic polymer (PVP) it is able to achieve faster drug release. It is one of the first attempts to reduce temperature and preparing immediate release tablet with talc as thermostable filler and matrix former [59].

Similar work also done by lower dose of Pantoprazole sodium (20 mg) processed below 100 ℃. Different polymer concentration tried but PEG 6000 and PVP K 12 found suitable for faster drug release. The porosity of tablet plays a vital role in it; infill percentage % can be lower down to 50% helps to reduce drug release up to 3 min. It is one of the futuristic approaches for thermo-sensitive drugs via this technique [60].

The drug paracetamol which is higher drug content requires different strategies to formulate tablet. It mainly needs excipients which equally contribute in formulation leads to increase the tablet weight. High drug loading paracetamol (80%) prepared using grind with PVP K-25 and CCS for the certain period of time. The cartridge was used to load it into the printer. The oval-shaped tablet is prepared so as to achieve drug release more than 90% within first 10 min. This effort can be utilized further in case of high dose formulation. This technique can fulfill all the needs of immediate release dosage form [61].

The study performed for identification of acceptable polymers suitable for 3D printed tablets with the help of FDM. The filament of drug-polymer was prepared by HME, Haloperidol was chosen as a basic drug with pH-dependent solubility in aqueous media. The crushed filament gives rapid drug release when it is compared with various set of polymers. For infill density it was observed that 60% infill was faster than 100% infill to achieve the drug release in case of immediate release dosage forms [62].

For inflammatory bowel disease treatment, the model drug chosen was 4-aminosalicylic acid and 5-aminosalicylic acid (aminosalicylate isomers). The available PVA filaments which incorporates the drug with the filaments were prepared by loading APIs onto PVA filaments. The drug loading was found to be 0.06% w/w and 0.25% w/w for 4-ASA and 5-ASA, respectively. The tablet prepared with 10.5 mm diameters for both the drugs. The infill percentage was in the 10%, 50% and 90% to check with release pattern. The infill percentage can correlate to the release profile, lower infill percentage can gives faster drug release pattern. Release profile mainly dependent on printing parameters that can easily modified as per the need. The higher temperature for extrusion is 210^0^C for PVA to print the filament. It is drawback of filament, whereas other polymeric filaments can easily print at the temperature below the decomposition temperature so that it will not degrade. In this case, 5-ASA was found to be the ideal drug for FDM process which melts at 278^0^ C but 4-ASA was found to be thermally degraded at 130 ℃, that was the major problem occurred in the process [31].

#### Geometry of tablets

The effect of geometry is one of the pioneer works which explored the possibilities with different shape of tablets and its effect on release pattern. The 3D printing is only possible methods which effectively formulate the shapes like cylinder, cube, torus, pyramid and sphere. It overcomes the boundaries of traditional methods and easily forms the design of required size and shape by using the software. Otherwise by the use of powder compaction method it is impossible to create different geometries. About 4% Paracetamol is loaded successfully with PVA filaments, from the release data it confirms that geometry plays a vital role. By keeping surface area constant the observed release pattern was (fastest first) pyramid > torus > cube > sphere > cylinder. Sometimes amount of drug loading percentage is higher needing some plasticizing agent for the proper flexibility and temperature while printing [32].

One of the novel concepts of channeled tablets is introduced in the tablet design. It helps to increase the surface area of the tablet with media perforation through its structure. The shorter channels give more efficient drug release than the longer channels. The size of ≥ 0.6 mm is essential to accelerate the drug release and meet pharmacopeial criterion for immediate release products. It is anticipated that a new generation of dosage form of complex geometry will emerge to tailormade drug release via controlling the media flow through these built-in channels [63].

In extended release, ellipse-shaped solid tablets were prepared by using prednisolone. Model drug used in the study because of its (melting point: 235 ℃) high thermal stability and neutral nature. It helps to test the filament ability of API content in the dose and its release before going in the nozzle of printer. Saturated methanolic solution of drug was used for loading due its drug solubilising ability and swelling of PVA without hampering the filament structure. The maximum drug loading is about 1.9% w/w; thermal analysis and PXRD study confirms the amorphous form within the PVA matrix. The FDM 3D printers can be exploited as a platform to construct flexible dose tablets from purpose built drug containing filaments for drug release over the 24 h [43].

#### Abuse deterrent formulations

Drug abuse is the biggest concern in the recent past. The opioid drugs are misused via oral way, nasal pathway or by making fine particles with directly or dissolved in solvents to intake. The metformin is used as model drug because of its aqueous and alcohol solubility similar to abuse drug oxycodone. The egg-shaped tablet is made in such a way that it will remain intact after mechanical force applied externally. Shape will not be easy to break as that of normal tablet. The various household and laboratory tools used to confirm the resistance of formulation but it found to be negative. Particles obtained are higher in particle size that it will not easily useful for snorting. PVA and sorbitol extrudes at 170 ℃. For immediate drug release ideal formulation was found to be 45% infill density and 15% w/w drug load. It is one of the essential needs to made abuse deterrent formulation for avoid its unethical use [64].

## Pediatric dosage form

Study was done for pediatric dosage forms in which it was difficult to deliver right dose at right time. In this case, baclofen drug was used along with polyvinyl alcohol with sorbitol for the preparation of 3D printed tablets by using FDM. The tailormade formulation was able to prepare with the help of quality by design approach. The successful attempt was made to overcome the problems like diversity and vulnerability in pediatric population [65].

## Multi-function tablet

### Compartments

The first-line anti-tuberculosis drugs isoniazid and rifampicin were successfully fabricated by 3D printing technology combined with hot melt extrusion of drug containing filaments. The separate compartments were made to slow down the release and absorption of drugs. Sealing of the compartment had a more prominent effect on the drug release kinetics than the spatial confinement of the unsealed compartment for the in vitro studies. The different compartments are made which are isolated from each other; it gives promising results [66].

### Dual release mechanism

Theophylline is chosen as the thermostable model drug which possesses high melting point 273 ℃. The 3D printed tablet is made in such a way that it will serve as both immediate and extended release tablets via HME 3D printing. The drug loaded filaments was made using cellulose-based polymers (hydroxypropyl cellulose SSL) and methacrylic based polymers (Eudragit RL, RS). The dose range for study lies within range of 60–300 mg, and it was possible to load the drug of high dose range. Sometimes high temperature of FDM 3D printing leads to degradation of thermolabile drugs and polymers. The use of polymers like Eudragit RL helps to release the drug in immediate manner and Eudragit RS gives extended release of the formulation. The optimum temperature printing temperature lies within the range of 220–260 ℃ which is much higher than the room temperature. The advantage for patients is that it is made in the caplet form which is easy for swallowing. The dose accuracy lies within range of 91–96%. By using HME processed pharmaceutical filament linear relationship between the mass and printed volume maintained and utilized for digitally controlling the dose (*R*^2^ = 0.9995) [40].

### Increment in drug loading capacity

For the 3D printing in drug formulation various strategies are used considering the physicochemical properties. Mainly FDM (Fused deposition modeling) is currently used for number of formulations with different % variation in drug loading. The different strategies have been reported for design of 3D printed drug delivery system using various types of drugs taking into account its physiochemical properties. Mostly with the help of fused deposition modeling (FDM) attempts are to increase the drug loading capacity. FDM provides the mechanically stable printed platform suitable for 3D printing, having high resolution (30–200 µm) and no further specific process required for the printed objects [40].

### Capsule-shaped tablets

The synthetic corticosteroid budesonide was chosen as the model drug used for the treatment of inflammatory bowel disease. The polyvinyl alcohol filaments were prepared in the caplets form capsule-shaped tablets by using 9 mg of budesonide filaments. Drug loading was less than 5% w/w due to sticking of drug to container, during processing of HME and irregular extrusion drug might be lost. The goal for budesonide modified release to increase duration of action, optimum therapeutic levels and reduction in the side effects at specific gut site. In the commercial formulations by the use of pH dependent coatings it is achieved in formulation. So to achieve the respective dissolution kinetics enteric coating is done by fluid bed coating for 3D printing caplets. The release pattern of 3D printed tablets is enough to use for the treatment of inflammatory bowel disease [67].

### Low melting point drug

In case of low melting point drug like ramipril (109 °C), it has been successfully demonstrated by FDM at 90 °C. The polymer shows no signs of drug degradation, the mixed polymer printlets showed slightly faster drug release compared with single polymer used. It shows selection of proper drug-excipients combination for FDM 3D printing can be overcome to the drug degradation problem which is due to thermal heating during the process [68].

In reported study filament with the API which dissolved in polymeric matrix by HME technology. The HPMC extruded filaments were printed with optimized 3D printing parameters which produced tablets with controlled release tablets having different 3D structures. It clearly demonstrates that coupling of FDM based 3D printing with HME offers a novel, economical and efficient method for manufacturing complex, personalized dosage forms and better controlled release profiles dosages that can be prepared as per requirement of individuals [69].

## Vascular regeneration

Blood vessels play a vital role in nutrients transportation and oxygen for different internal organs. Blood which is connective tissue plays a vital role in functioning of the organs. In case of trauma and diseased conditions, major vasculatures are interrupted or damaged make difficulty while exchanging nutrients and gases among the organs. In 3D printing gives hope in the vessels regeneration as tissue engineering becomes easier. In advanced tissue engineering technology in the regeneration of blood vessels in addition to endothelial cells and muscle. Polycaprolactone (PCL) and methacrylate gelatin polymers mainly used in this case [70]. Method of electrospinning was used with polycaprolactone so as to improve adhesion property and elastic characteristics. This framework with gelatin polymer organized in linear pattern with muscle cells by using a rotator bioprinter. This model synergistically added benefits of two technologies and effective as new strategy for vascular tissues modeling [70].

## Fabricating materials

Collagen which is a prime protein structure used in 3D printing. The regeneration of cartilage and rigid connective tissues are designed by using collagen. Camptothecin loaded in chitosan polymeric micelles by customized by using 3D printing technology [71]. This formulation was protected from gastro-intestinal tract by using enteric coating polymer so as to bypass the harsh environment. The combination therapy of 3D printing and nanomedicine together improved the drug absorption in the intestine; it protects the drug from an acidic environment and prevents indignity [71].

In the parallel scenario many synthetic polymers recognized for its use in 3D printing technology. Polycaprolactone (PCL) is mechanically strong enough to process via 3D printing technology for specific models. In one attempt magnesium-polycaprolactone loaded with melatonin prepared 3D structure. It is hypothesizes to be used in the treatment of osteosarcoma i.e. bone cancer. This experiment helps to analyze 3D printed drug model helps in giving anticancer effect. It is a novel approach to use in the treatment of osteosarcoma [72].

## Dermal regeneration

The skin comprises of hypodermis (adipose tissue), dermis (vascularized layer) and epidermis (outermost layer) which covers the maximum surface of the human body. The skin which is largest organ in the human body gives the protection against mechanical shock and helps to maintain thermal homeostasis [73]. Skin regeneration can be major back holds permanently. Regeneration of skin, improper vascular network and insufficient angiogenesis stimulation were the main challenging factors [74].

The induction of angiogenesis were the primary focus of the researchers, microstructure of strontium silicate were generated and embedded in bioink polymer. The cell writing 3D printing technique was used to model the vascularized tissue exhibited the angiogenic activity in both in vivo and in vitro cases. It provided the path for tissue engineering of vascularized skin which possess faster recovery rate [74].

## COVID essentials

In a worldwide outbreak of COVID-19 the virus mainly affected the respiratory passage. The insufficient supply of N 95 masks, personal protective kits, testing kits, face shields were an essential step for controlling the growth of virus in wider range of people. 3D printing helps in a pandemic to fabricate these materials with rapid rate and efficient way to meet the needs of needy patients [75]. Polymers such as polycarbonate, polyvinyl chloride and polyester are generally used for face shields preparation. 3D laser scanning is used in the development of N95 masks. In case of medications useful for COVID control like ritonavir, lopinavir, chloroquine and hydroxychloroquine pills were prepared by the layer by layer deposition of drugs by using fused filament, powder extrusion and ink jet technology [73, 77]. The different drug-polymer combinations with novelty are described in the 3D printing formulation in the Table 1.

### 3DP special characterization of filaments

Novel technique recently explored in the pharmaceuticals for acceptance by regulatory agencies. Apart from the general criteria special characterization done for the 3D printed pharmaceutical products are as follows:

1. *Three point bend test* The capacity of filament is studied for withhold limit within specified time. It is testing of filament for its brittleness, flexibility and stiffness. It helps to understand the strength of filament suitability for 3D printing.
2. *Feedability test* Specific for FDM, initial test for filaments before printing to check properly the extrude filament from the tip of nozzle. The temperature set for printing must be compatible with filament to extrude easily through pointed tip of the nozzle.
3. *Texture Analysis (TA) Screening Test* Test is performed for the capacity to withstand filaments pass into the 3D printer of any type. It must possess certain strength in filament. During the test, equipment of texture analyzer carry load of 5 kg and small part of filament passed through it. It shows the capacity of filament to carry certain amount of load [78].

### Clinical practice

3D printing is developing technology with high accuracy, easy to process and affordable to everyone but clinically it will take time to incorporate in the practice. The 3D printed products are still in developing stage and regulatory framework is not well established, the product for clinical practice is less in number. Adaptation of 3D printing is still less for clinical practice. To prove clinical efficacy in 3D printing dosage form clinical practice should be more in practice. For the tailormade formulation 3D printing can be considered to deliver instantly the medication by the medical practitioner. It will provide the need of instant therapeutic delivery by personalized medication. It will also help to regulate 3DP practice clinically [79, 80].

As per the need of patient, drug loaded filament can be made for desired personalized product. It also gives an aesthetic purpose by using 3D printed formulation in comparison with the traditional market. Considering the current standards like friability cannot be directly considered for 3D printing parameter. The friability limit which is 1% acceptable limit cannot be directly considered for 3DP. Depending upon the class of patient other characterization also varies as per the need of clinical practice [81, 82]. The guidance given by the FDA on Technical Considerations for Additive Manufactured Medical Devices on the December 2017, helps to understand the need of 3DP [83].

For consideration in the clinical practice size, shape and percentage of infill is well defined to meet with patient specific criteria [83]. In the current status of 3D printing, good manufacturing practice (GMP) is not complies with all well-established parameters. These parameters must be well understood to prepare dosage form within time limit and cost involved for it. So, to ensure the validation, safety must be main concern for considering its use for human in future [84].

In case of futuristic approach with 3D printing, clinical practice must be congruent with manufacturers, scientist and regulatory authorities. Considering the fact regulations are not well developed, as time evolved product will accept by the authority to consider in the actual practice. FDA by giving first approval to 3D printing technology it will develops standards in upcoming years for clinical practice. The Center for Drug Evaluation and Research Office of Pharmaceutical Quality takes effort in the investigation of 3DP into clinical practice. This factors need to be consider in approach towards human consumption of 3D printed product. Such initiative from all stakeholders of pharmaceutical industry helps to enhance quality of practice for the futuristic way forward to the personalized medication [82].

### Regulatory perspectives

After the first FDA approved 3D printed product in the year 2015 named Spritam® (levetiracetam, antiepileptic drug) by Aprecia Pharmaceuticals Co. [85], OsteoFab® (Orthodontic implant) by Oxford Performance Materials Inc. [86], Unite 3D™ Bridge Fixation System by Zimmer Biomet Holdings and 85 other products initiated developing different drug products and medical devices by this novel tool. The device must be equivalent as per the 510(k) of FDA. The device is compatible to use if it has proven the equivalency with same characteristics in technology, intention of use, safety and market potential. The equivalent device with same characteristics but with different technological characteristics without any safety and efficacy affected.

The next generation impact technology getting rapid attention among pharma peers increases the competition among them. By considering progress, FDA in December 2017 released ‘Technical Considerations for Additive manufacturing of Medical Devices’ which gives potential regulatory insights, for the current thinking of the agency. Also key chemistry, manufacturing and control are the requirements for the approval of 3D-printed drug products and medical devices [83].

Particularly in case of pharmaceutical tablet development, formulation aspects of quality control specifications and requirements related to printer is not clearly mentioned. The availability of printer in the market having variation in their features is high, different technical aspects like software, hardware and printing capacity is highly differ from each other. Regulation with respect to formulation and development of dosage form needs clear idea to avoid discrepancy in the quality of product. In case of pediatric patients, palatability and ease of swallowing is important to administer proper dose. The ideal pediatric product must have dose flexibility with patients of age group 0–17 years so that single dosage form can easily give to patients [87].

Succeeding with first approved product FDA comes out with the Guidance Agenda 2016 for its regulatory report inculcated for 3D printing in pharmaceuticals. For NDA approval new drug and devices should be safe and effective in its efficiency. The device must be equivalent as per the 510(k) of FDA. The device is compatible to use if it has proven the equivalency with same characteristics in technology, intention of use, safety and market potential. The equivalent device with same characteristics but with different technological characteristics without any safety and efficacy affected. 3D printing is prepared for the need of patients in the specific concerned criteria like multiple dosages in unit dosage form. 3DP manufacturing in pharmacy and hospital setting are exempted from section 501(a)(2)(B)(concerning cGMP requirements), section 502(f)(1) (concerning the labeling of drugs with adequate directions for use) and section 505 (concerning the approval of drugs under new drug applications or abbreviated new drug applications) of Food, Drugs and Cosmetic Act (FD&C Act) [88]. Moreover for the exemptions from 501(a) (2) (B), 502(f) (1) and 505 of the FD&C Act, it must meet criteria listed in the acts [89]. 3D printed drug product must also be manufactured in accordance with current CMC standards as set forth in the 21 CFR 200 s & 300 s and other relevant guidance for 505 b (1), 505 b (2) type of submission. Personalized drug products may not align with any of these pathways-505(b) (1), 505(b) (2) or 505(j) because products submitted by these pathways need to have consistent quality.

3D printing is more for class than the masses. It is more prone to have special application in the particular sections of patients such as pediatric, geriatric and particular diseased conditions. Usually dose is calculated according to patients need and dispensed with respect to prescribed dose by the pharmacist. But FDA is more concern about dose accuracy, efficacy, stability, safety, pharmacodynamic and pharmacokinetics effect [90].

### Cost effectiveness

By considering growth of 3D printing in different sectors like electronics, automotive and healthcare industries cost can be important factor for its sustainable growth. Comparing to traditional manufacturing 3D printing cost depends on the need of patients. The need of dose for patient is smaller hence ultimately it will reflect into cost of pharmaceuticals dosage and vice-versa. By choosing raw materials in small amount and bypass number of steps involved in traditional manner cost can be reduced and affordable to everyone. In conventional manner where multi-functional tablets are not easy to formulate but in case of 3D printing it is facile method to accept. Sometimes it was observed that well known conventional tablet is more than sufficient for emergency need for patient. Considering economical 3D printed product it will evolve with time and accepted for application in pharmaceuticals [91].

### Future perspectives

The 3D printing revolves as the new dawn in the field of pharmaceuticals. The upcoming era is of personalized medication. Depending upon the need of patients accurate dosing can be done at respective home, pharmacy centre and pharmaceutical industry [92]. The growth of 3D printing industry is increasing in the exponential manner since it is gaining huge market demand. The estimation of 3D printing healthcare market size in global scenario is to reach $3692 million by 2026, considering compound annual growth rate (CAGR) of 18.2% from the year 2019 to 2026.

Many academic institutions and manufacturers started installing 3D printer for the instant preparation of dosage forms and medical devices. It has the benefit of inclusion of multiple drugs in the unit dosage form which administer prescribed medicine with highly efficient manner. It also provides medicines in the affordable price because of its fewer requirements of additives and machinery. Highest safety incorporation of multiple drugs in a single dosage form will ensure timely administration of entire prescribed medicine with highest safety and least toxicity. This will ensure efficient, cheap and timely availability of healthcare services to the patients. Manufacturing products on demand is likely to reduce the overall investment of institutions in healthcare products. The newer methods are able to evolve for personalization of medicines (pharmacogenomics). It can able to change the characteristics of dosage form such as release profile as per the need of patients. Currently available 3D printed formulations are patented comparing to other traditional marketed formulations hence they are highly expensive. In the broader perspectives personalized medicines will be safe and accurate mode in the future. Perhaps in future prescription is given after studying history and current diseased conditions in pharmacy and dose is given by 3D printer as per need.

3D printing formulation can be made as target drug product profile by complying with all regulatory guidelines. The understanding of real example becomes easier with the help of 3D printing models. Overall, this technology will be paradigm shift in the pharmaceuticals and important part of day-to-day life to bring ease and comfort.

## Conclusion

The 3DP is an emerging technology; it could change the overall scenario of mass production for dosage form to tailormade personalized dosage formulation on demand. The recently FDA approved 3D printed tablet Spritam® was a game changer in the history of 3D printing technology. By including HME process, the cost is higher but 3D printers cost is quite low that is easily compensate with each other and makes cost of production bearable. It is expected to evolve rapidly in the upcoming decade to provide the novel manufacturing process. Also HME filaments have advantages over the 3D printed materials in the parameters like less loss of materials, compact size and ease in the processes. The new benchmark is set in the tailormade formulations which are beneficial for clinical studies to personalized medicines. The researcher expected to carry out different outcomes and possibilities that 3DP can bring. In products such as low dose, potent APIs and orphan drugs can be formulated with high accuracy and precision. The regulatory requirements need to bring different perspectives for its efficient use in the actual practice. As it complies with all respective standards, it will become easier for regulatory agencies to bring 3DP product into the market.

## Data Availability

Data sharing is not applicable to this article as no datasets were generated or analyzed during the current study.

## Abbreviations

3DP: Three-dimensional printing
FDA: Food and drug administration
FDM: Fused deposition modeling
cGMP: Current good manufacturing practices
HME: Hot melt extrusion
API: Active Pharmaceutical Ingredient
PXRD: Powder X-ray diffraction
PE: Piezoelectric
TIJ: Thermal inkjet
SLA: Stereolithography
Tg: Glass transition temperature
SLS: Selective laser sintering
4-ASA: 4-Aminosalicylic acid
5-ASA: 5-Aminosalicylic acid
mg: Milligrams
kHz: Kilohertz
pL: Picolitre
μL: Microlitre
μg: Micrograms
μs: Microseconds
ms: Milliseconds
PAM: Pressure-Assisted Microsyringes
ABS: Acrylonitrile butadiene styrene
PVA: Polyvinyl alcohol
PEO: Polyethylene oxide
HPMC: Hydroxypropyl methylcellulose
PVP: Polyvinyl pyrrolidone
RSD: Relative standard deviations
UV: Ultraviolet
PCL: Polycaprolactone
PLA: Polylactic acid
PGA: Polyglycolic acid
PPS: Polyphenylene sulfide
PP: Polypropylene
PEG: Polyethylene glycol
PET: Polyethylene terephthalate
PLGA: Polylactic-coglycolic acid
nHA: Nano hydroxyapatite
CCS: Croscarmellose sodium
NDA: New drug application
DEF: Diethyl fumarate
PPF: Poly propylene fumarate
pHEMA: Poly (2-hydroxyethyl methacrylate)
DMA: Dimethacrylate
PEG: Poly ethylene glycol
PEGDA: Polyethylene glycol diacrylate
